# The predictive values of state and trait mindfulness on emotion understanding in primary school children

**DOI:** 10.3389/fpsyg.2026.1849238

**Published:** 2026-07-10

**Authors:** Katharina Voltmer, Annette M. Klein

**Affiliations:** 1 Institute of Educational Science, Leuphana University Lüneburg, Lüneburg, Germany; 2 International Psychoanalytic University Berlin, Berlin, Germany

**Keywords:** breath-based state mindfulness, children, emotion understanding, social–emotional competence, trait mindfulness

## Abstract

**Introduction:**

Although positive effects of mindfulness-based interventions in schools on children’s cognition and behaviors are frequently reported, there is a lack of studies on the association between children’s mindfulness and their interpersonal social-emotional competences. The present study addresses this gap by analyzing the association between breath-based state mindfulness, self-reported trait mindfulness, and emotion understanding in German primary school children.

**Methods:**

Breath-based state mindfulness, self-reported trait mindfulness, and emotion understanding were assessed using a breath-counting task, the Mindful Attention Awareness Scale for Children, and the Adaptive Test of Emotion Knowledge, respectively, in *N* = 66 third-grade children (mean age = 8.52 years, *SD* = 0.69, 48% female) across three measurement points over an eight-months period during one school year. The effects of children’s mindfulness on their emotion understanding across all measurement points were analyzed in a multilevel mixed-effects linear model, controlling for gender, working memory updating, and concentration performance.

**Results:**

In addition to the significant effects of time and children’s working memory updating, a significant effect of breath-based state mindfulness on children’s emotion understanding was found.

**Discussion:**

These findings may suggest that children’s mindfulness practice could have positive effects on social-emotional competences, such as emotion understanding, even when explicit components of social-emotional learning are not included in the mindfulness practice. This study highlights the potential relevance of brief mindfulness practices for supporting children’s social-emotional development in school contexts. Future research should further investigate this association and potential mediators in more detail using larger samples.

## Introduction

In recent years mindfulness-based interventions (MBIs) became increasingly popular in the educational context (Porter et al., 2022). In primary schools as well as in high schools MBIs were conducted with children and adolescents and their effects on different outcomes have been evaluated. Meta-analytic research and systematic reviews showed that MBIs can have a positive impact on children’s externalizing and internalizing symptoms (i.e., attention problems/ADHD behaviors, conduct problems, and anxiety), emotion regulation, perceived stress, prosocial behavior, resilience, executive function, attention, and mindfulness (Phan et al., 2022; Segal et al., 2021). However, concerns have been raised that the implementation of MBIs in educational settings may place a disproportionate emphasis on individual self-regulation and self-improvement skills, while paying less attention to ethical, relational, and pro-social dimensions of education (Berger et al., 2024). With this in mind, it is important to examine the extent to which MBIs in schools also promote social-emotional skills, which have repeatedly been confirmed as predictors of both academic and social success in school and beyond. In the present study we therefore aim to examine the association between mindfulness in primary school children and a key social-emotional skill, namely the ability to understand others’ emotions.

### Mindfulness

Mindfulness has been defined as “the awareness that emerges through paying attention on purpose, in the present moment, and non-judgmentally to the unfolding of experience moment by moment” (Kabat-Zinn, 2003, p. 145). Thus, it is the ability to pay sustained attention and be present to all kinds of experience. There is both theoretical and empirical evidence that trait mindfulness and state mindfulness are two distinct constructs that require separate operational definitions and measurement tools. While trait mindfulness measures assess a person’s stable characteristic or enduring behavioral pattern (Kiken et al., 2015), state mindfulness measures evaluate the degree to which someone engages in mindfulness practices at a particular time (Medvedev et al., 2017). Previous studies in adults have found only limited overlap between trait and state mindfulness (Thompson and Waltz, 2008), while among individuals with meditation experience, stronger associations between trait mindfulness and facets of state mindfulness were observed with increasing frequency of meditation practice (Bravo et al., 2018).

In young children, MBIs address, for example awareness of breath and body sensations, sounds, movement, thoughts and emotions (Jackman et al., 2019). Thus, when being mindful, children’s awareness can be directed toward internal (feelings, thoughts, etc.) or external (visual, auditory sensations, etc.) experiences. These attentional and awareness-related processes may be particularly relevant for children’s social-emotional functioning, as recognizing and interpreting emotional states in oneself and others require sustained attention to internal and external emotional cues. Thus, the literature suggests that mindfulness (in childhood) may involve both relatively stable dispositional tendencies and more situational attentional states that may require distinct operationalizations and measurement approaches.

However, according to a current systematic review, for children and adolescents primarily self-report questionnaires are available that assess trait mindfulness (Bender et al., 2023). Besides questionnaires that measure trait mindfulness as a unidimensional construct (e.g., the Mindful Attention Awareness Scale-Children; Lawlor et al., 2014), other instruments assess multiple facets of trait mindfulness by differentiating, for example, between observing, acting with awareness, and accepting without judgment. These include measures such as the Child and Adolescent Mindfulness Measure (Greco et al., 2011; Mezo et al., 2020). However, self-reports have limitations such as biases in self-perception which might be especially problematic in mindfulness research. For example, results could be distorted by socially desirable response behavior, as a study by Brown and Ryan (2003) showed. Even more serious, however, is the problem that items in mindfulness questionnaires are interpreted differently by people with different experiences in theory and practice of mindfulness (differential item functioning). This was indicated by a study by de Bruin et al. (2013), in which adolescents with more yoga and meditation experience achieved significantly lower scores in a mindfulness questionnaire than adolescents without this experience, although these practices are expected to promote mindfulness. Paradoxically, people with low levels of mindfulness in particular might lack the metacognitive ability to notice their own lack of mindfulness, which means that it is overestimated in self-report procedures (Grossman, 2019; Sauer et al., 2013). Therefore, researchers have recommended also using other methods to assess trait mindfulness and including objective state mindfulness measures (Bender et al., 2023; Goodman et al., 2017).

### Breath-based mindfulness

Addressing this recommendation, Levinson et al. (2014) developed and validated breath counting as a behavioral measure of state mindfulness in adults. In this task, participants count their breaths. For each of the first eight breaths, participants are asked to press a specific key on the computer keyboard. For the ninth breath, they are asked to press a different key and then repeat this process until the time is up after 18 min. They also follow a rule when they notice mind wandering, i.e., that they miscounted their breath. We consider this task a behavioral indicator of state mindfulness because it requires sustained present-moment attention to the ongoing experience of breathing as well as the recognition of attentional lapses and mind wandering over an extended period of time during a meditation-like cognitive task. This conceptualization is consistent with influential operational definitions of mindfulness emphasizing the self-regulation of attention and awareness of present-moment experience (Bishop et al., 2004). Breath-focused attention is central to many mindfulness practices because the breath serves as a stable anchor for present-moment awareness. Accordingly, successful performance in the breath counting task requires maintaining attention on the breath, noticing when the attention drifts away from the task, and intentionally redirecting the focus back to the breathing process. Levinson et al. (2014) have argued that these processes closely correspond to core attentional and meta-awareness components of mindfulness and has provided evidence that breath counting accuracy is associated with greater meta-awareness and reduced mind wandering. At the same time, we acknowledge that breath counting performance is unlikely to reflect mindfulness exclusively, as the task may also involve cognitive processes such as sustained attention, working memory, and general task engagement. Recently, Zhuang et al. (2025) investigated whether the breath counting task can serve as a valid behavioral measure of mindfulness in 9–13-year-old children. They reported psychometric properties and response patterns similar to those observed in adults and concluded that breath counting may be a promising method for assessing mindfulness in children. However, children’s breath counting performance was unrelated to their self-reported mindfulness, in contrast to the weak but significant correlations reported in adults (Levinson et al., 2014).

Since our study focused on a younger age group than that investigated by Zhuang et al. (2025), the task may require further developmental adaptation. When presented in a more child-friendly format and with a shorter duration, it could serve as an objective indicator of breath-based state mindfulness in young children.

### Emotion understanding

In the context of this study, the term emotion understanding refers to the knowledge and understanding of the emotions of other people. This includes the recognition of emotional facial expressions, the understanding of external and internal causes of emotions and the knowledge of display rules and about the application of various functional and appropriate emotion regulation strategies.

The relevance of children’s emotion understanding in their daily social interactions is illustrated by the model of social–emotional learning (SEL; Collaborative for Academic, Social, and Emotional Learning [CASEL], 2015). According to this model, five interrelated areas of competence form the basis for achieving intra- and interpersonal goals across different contexts and thus lead to effective social interactions. These areas of competence are (1) Self Awareness, (2) Self-Management, (3) Responsible Decision Making, (4) Relationship Skills, and (5) Social Awareness. They influence the experience of self-efficacy, but also relationships with peers and adults. Children who have these skills can manage their social interactions more effectively across different contexts, i.e., in a wide variety of situations with peers, parents and teachers. According to the model, emotion understanding is an important aspect of social awareness and relationship skills.

Consistent with this, a meta-analysis has shown cross-sectional and longitudinal associations between children’s emotion understanding and their social skills, peer acceptance, and academic performance (Voltmer and von Salisch, 2017). The fact that emotion understanding relies on a strong working memory and well-developed receptive language skills – particularly in complex social interactions such as those encountered at school – may represent underlying mechanisms for its association with academic success (Morra et al., 2011; Tang et al., 2021). In addition, empirical evidence indicates that young children with low emotion understanding scores were later observed to exhibit less regulated and more angry and aggressive behavior in the classroom (Denham et al., 2002). This suggests that children with more advanced emotion understanding know better when and how to regulate their emotions (Miller et al., 2006). This is supported by another study in which emotion regulation mediated the relation between emotion understanding and adjustment in preschoolers (Di Maggio et al., 2016). In this context, mindfulness has also been linked to emotion regulation in children, suggesting a potential association between mindfulness and emotion understanding. Therefore, the present study focuses on emotion understanding as a key variable.

### Mindfulness and emotion understanding

While many studies exist on the connection between mindfulness and self-focused emotional competences like awareness of own emotions or emotion regulation (Cooper et al., 2018; Guendelman et al., 2017), little is known about the relation between mindfulness and social or social-emotional variables like prosocial behavior, peer relationships, or understanding other’s emotions, especially in children and adolescents. A literature review on the link between mindfulness-based practices in schools and the SEL framework concluded that the concepts of mindfulness-based practices and SEL are aligned, but that the different SEL competency domains are assessed at different frequencies in MBIs (Feuerborn and Gueldner, 2019). The self-management domain of SEL was assessed in all studies that were reviewed, whereas the social awareness and social relationship skills domains were present in only a minority of studies. Schonert-Reichl et al. (2015) reported positive effects of their SEL program including mindfulness (MindUp™) in fourth and fifth grade in Canada on (among others) empathy, perspective taking, pro-sociality, and peer-acceptance. The same mindfulness program was used and evaluated with Ugandan children and the results showed an increase in empathic behavior and a decrease in hostility (Matsuba et al., 2021). Sciutto et al. (2021) investigated the effects of a mindfulness curriculum which was a mixture of MindUp™ and another program in Pennsylvania, and also found positive effects on the children’s prosocial behavior. Another mindfulness-based program, which was applied in a sample of kindergarten and primary school children, lead to increases in children’s emotion recognition skills (Thierry et al., 2022). It should be noted, however, that all these programs were not exclusively mindfulness programs, but explicitly included units on perspective-taking and prosocial behavior. Only a few other studies included analyses on the association between mindfulness and social–emotional competences in their studies without conducting an MBI: Rodríguez-Ledo et al. (2018) studied the association between trait mindfulness and emotional intelligence in a Spanish speaking sample of 11- to 15-year-olds. They found significant correlations between external (e.g., attention to sounds, observations) and internal (e.g., attention to thoughts, feelings, etc.) mindfulness and general empathy (*r* = 0.205; *r* = 0.231) but not between external and internal mindfulness and interpersonal emotional intelligence (*r* = 0.138; *r* = 0.144). In a study by Mestre et al. (2019), examining the association between trait mindfulness and trait emotional intelligence in a sample of *N* = 318 Spanish children and adolescents aged 8–16, no relation was found between mindfulness and the perceiving and understanding emotions subscale of emotional intelligence. The same applied to the study by Foster et al. (2018) in their study on *N* = 108 Australian adolescents in eighth grade.

Overall, the existing literature provides only limited and partly inconsistent evidence regarding the association between mindfulness and children’s social–emotional competences, particularly emotion understanding. In addition, prior research has relied almost exclusively on self-report measures and has assessed only trait mindfulness. None of the mentioned studies included self-report or behavioral measures of state mindfulness, and only one study used an objective test of social-emotional skills, i.e., emotion recognition (Thierry et al., 2022).

### The current study

The present study aims to address these limitations by examining the relationship between breath-based mindfulness – a behavioral indicator of state mindfulness – and self-reported trait mindfulness with children’s emotion understanding, as an indicator of social awareness and social relationship skills. Based on Feuerborn and Gueldner’s (2019) notion that the concepts of mindfulness-based practices and SEL are aligned, we expect that children’s breath-based state mindfulness and trait mindfulness are positively associated with emotion understanding. Given that state and trait mindfulness have been shown to be distinct constructs in adults, we expect both measures to contribute independently to children’s emotion understanding. Thus, for the present study in which the children’s performances are aggregated over three measurement points, we expect that children’s breath-based state mindfulness, assessed with a more child-friendly version of the Breath Counting Task, and their self-reported trait mindfulness significantly and positively predict their emotion understanding in a multilevel mixed-effects linear model while controlling for known cognitive and socio-demographic variables.

We use data from the Breathing Break intervention project and address the research question with a sample of primary school children from northern Germany. In this randomized controlled study, classrooms and the respective teachers were either assigned to an MBI or an active control group. In the MBI group, the teachers received a short mindfulness-based self-regulation training and then carried out an intervention in the form of three- to five-minute mindful breathing breaks in their classrooms up to three times a day over a period of 9–10 weeks. In the active control group, the teachers had the children color mandalas for 3–5 min up to three times a day over the same period. Data were collected at pre-, post-, and follow-up assessments. In previous analyses of the dataset, data showed effects of the breathing breaks in terms of girls’ increased ratings of pro-sociality and maintaining of the quality of peer relationships during the COVID-19 pandemic in the intervention group, while those in the active control group decreased (von Salisch and Voltmer, 2023). The present study represents a secondary analysis of the Breathing Break dataset and does not focus on intervention effects or group differences between the MBI and the active control condition. Instead, the study exclusively examines correlational associations between breath-based state mindfulness, self-reported trait mindfulness, and emotion understanding across all participating children. For this reason, intervention-specific implementation details are kept brief in the present manuscript. Detailed information on the intervention, including the implementation of the breathing breaks and a detailed description of the original sample of children and teachers, the teacher training procedures, and the intervention and control group conditions, is provided in von Salisch and Voltmer (2023) and Voltmer et al. (2023).

### Control variables

Working memory updating performance and concentration are known correlates of both emotion understanding (e.g., Morra et al., 2011) and mindfulness (e.g., Li et al., 2021). These cognitive skills will therefore be included in the main analysis as control variables. On the children’s demographic level, age is an important predictor of emotion understanding because this competence develops rapidly over time in early and middle childhood. Although the age variance in the present sample is relatively low, the present study will control children’s age in the main analysis. Results regarding gender differences in emotion understanding are mixed. While studies of overall emotion understanding indicate that boys and girls do not differ in their competence (Fidalgo et al., 2018; Kårstad et al., 2015; von Salisch et al., 2017), analyses of individual components of emotion understanding have revealed differences in favor of girls in several studies (Bosacki and Moore, 2004; Fidalgo et al., 2018; Garner and Waajid, 2012; Kuhnert et al., 2017). Gender will be included in the main analysis if preliminary analyses reveal gender differences in emotion understanding within the present sample. Dual language learners (DLLs) and children from socially disadvantaged families often demonstrate lower levels of proficiency in tests of emotion understanding than children who grow up in monolingual families and those from middle-class families (Garner and Waajid, 2012; Kårstad et al., 2015). Therefore, this study will include children’s DLL status and socioeconomic background, i.e., their parents’ educational level, as control variables in the main analysis, if initial analyses identify significant associations with emotion understanding. Finally, group affiliation (intervention/control) will only be included as a control variable if initial analyses reveal significant group differences in emotion understanding, breath-based state mindfulness, or trait mindfulness at any measurement point.

## Method

### Sample

For the present analyses, data of a subsample of the Breathing Breaks intervention study of *N* = 66 children from 6 classrooms were used, because only these children completed the full version of the Adaptive Test of Emotion Knowledge for 3- to 9-year-olds (ATEM 3–9; Voltmer and von Salisch, 2021), consisting of 35 items (the remaining participants completed a shortened 15-item version of the ATEM 3–9). The mean age of the children was 8.52 years (SD = 0.69) at the first measurement point (T1) and 48% were female. Of these 66 children, 17% were DLL, 83% reported to only speak German. According to the parent questionnaires, which were completed for *n* = 63 of the children, 81% of the children lived in households in which at least one parent had a vocational qualification (vocational training, technical college, or university degree), whereas in 19% of the cases, neither parent had a vocational qualification. Data on parent’s vocational qualification and children’s self-reports of the languages spoken at home (collected as part of the ATEM 3–9) were strongly associated (Cramer’s *V* = 0.52, Fisher’s exact test: *p* < 0.001).

### Procedure

After receiving approval from the Ethics Review Board (Beirat für Ethikfragen in der Forschung) of the first authors university on May 6, 2020, that all research was performed in accordance with relevant guidelines and regulations, classrooms were randomly assigned to the intervention group (5 classrooms of the total sample) and the control group (4 classrooms of the total sample). T1 data collection took place in September 2020, T2 data were collected in December 2020, and T3 assessment took place in May 2021. Thus, the interval between T1 and T2 was approximately 3 months, and the interval between T2 and T3 was approximately 5 months (8 months between T1 and T3 overall). Due to the COVID-19 pandemic, children’s school attendance was limited between T2 and T3, and classes were split into two groups at T3. Data were collected using tablet computers and headphones. The emotion understanding test and the trait mindfulness questionnaire were administered in the classroom. Trained undergraduate research assistants assisted children who had technical difficulties with the tablets. The breath counting task, the working memory updating task, and the concentration test were completed in small groups of four to six children, each supervised by a trained undergraduate student in a separate room. Data collection in total (including instruments that are not evaluated in the present analysis) took three to four teaching hours at each measurement point, with the usual breaks in between. Because groups were split at T3, data were collected across 2 days, with one group of students assessed on each day. All methods were performed in accordance with the relevant guidelines and regulations. Informed consent was obtained from all children’s legal guardians. Along with the consent form, they completed a short questionnaire about their family situation, the language(s) spoken at home, and their educational and occupational background. Participation was voluntary for the children. Children who were not allowed or did not want to participate were either supervised in another room or quietly engaged with materials provided by the teacher. At each measurement point, all children received a small gift at the end of the data collection session.

### Instruments

#### Breath-based state mindfulness

At each measurement point, a breath counting task was used to assess participants’ ability to sustain attention on their breathing over an extended period of time. This task was derived from the Breath Counting Task for adults by Levinson et al. (2014), in which the test subjects are asked to count their breaths (combination of inhale and exhale) over a certain period of time and press a button on the computer for each breath. For the present study, the duration of the exercise was reduced from the original 18 min to 5 min, and the counting intervals were shortened from nine to five in order to ensure age-appropriateness. For each breath from one to four, a key should be pressed on the tablet keyboard, and for every fifth breath, a different key should be pressed. Children were asked to press the space bar and start counting again if they recognized that they miscounted. Children did not receive any feedback on whether their responses were correct or not. The children were instructed to breathe as naturally as possible, that is, neither very deeply nor very shallowly, and neither very slowly nor very quickly. Before starting the actual task, this procedure was practiced over a period of 1 min. A picture of a sky with clouds appeared on the tablet screen during the exercise and the test. Since children completed varying numbers of breaths or counting cycles within the five-minute period, breath-based state mindfulness was evaluated using the percentage of correct counting cycles (4x one key, 1x another key) relative to the total number of cycles completed. This measure was only included if the child’s average cycle duration fell within a plausible range of 7–25 s. Higher rates of correct counts were interpreted as indicating higher levels of breath-based state mindfulness.

#### Trait mindfulness

Children’s trait mindfulness was assessed at each measurement point via self-report using the Mindfulness Attention and Awareness Scale Adapted for Children (MAAS-C) (Lawlor et al., 2014). The items of the English version were translated into German using DeepL.com and subsequently adapted by the first author to ensure that the original meanings were fully preserved. The questionnaire consisted of 15 items, each rated on a 6-point Likert scale ranging from “almost never” (5) to “almost always” (0). An example item is “I rush through activities without being really attentive to them.” Across all items, children can achieve a total score ranging from 0 to 75. The items are reverse-coded so that higher scores indicate greater mindfulness. At T1, the MAAS-C achieved an acceptable internal consistency of *α* = 0.78.

#### Emotion understanding

At each measurement point, emotion understanding was measured using the ATEM 3–9 (Voltmer and von Salisch, 2021). It consists of 35 items that assess the following seven components of emotion understanding: (1) Recognizing emotions in faces (Recognition; 6 items), (2) Identifying emotions in situations (Situations; 6 items), (3) Identifying emotions from desires (Desires; 5 items), (4) Identifying mixed emotions (Mixed Emotions; 5 items), (5) identifying emotions from (false) beliefs (Beliefs; 5 items), (6) distinguishing between shown and felt emotions (Felt/Shown Emotions; 5 items), and (7) knowledge of emotion regulation strategies (Emotion Regulation; 3 items). The items are arranged to form a child-friendly story, and the difficulty of the items increases within and between components as the story progresses. The items are independent of each other in terms of content, so that by using termination rules, only those items are presented in each component that matches the child’s ability level. The ATEM 3–9 was administered on tablets with gender-specific audio output and gender-specific child protagonists in the story. For the most items, children responded by tapping on one of four faces with pictures of facial expressions of anger, sadness, fear, happiness, disgust, or surprise. Items on emotion regulation strategies required children to tap on pictorial representations of behaviors or thoughts of the protagonists. Children received one point for each correctly answered item, with a maximum score of 35 points. Items that were not completed due to the application of termination rules were scored zero points. The EAP/PV reliabilities (which can be interpreted as Cronbach’s alpha) of the seven components ranged from 0.82 to 0.92 in the norming sample that includes the present data (Voltmer and von Salisch, 2021).

#### Working memory updating

At each measurement point, children’s working memory updating was assessed through the app version of the backward digit span task of the Eichstätter Messung des Arbeitsgedächtnisses (EI-MAG; Eichstatt Measurement of Working Memory) (Oesterlen et al., 2018). A sequence of digits that ranged between 1 and 9 were presented auditorily via headphones at intervals of 1.5 s. A 3×3 number pad containing the digits 1 to 9 then appeared on the tablet screen. Children were instructed to tap the digits of the previously heard sequence in reverse order. The number of digits to be memorized increased as the test progressed, thus increasing the level of difficulty. Children had to correctly reproduce two of the three series of the same length in each block before they could move on to the next level. The easiest block required children to reproduce two digits in reverse order, whereas in the most difficult block, a series of eight digits had to be reproduced in reverse order. The practice phase preceded the test phase. The test ended after three errors within a block. The number of correct answers was used for the analyses. Children who did not provide any correct answers in the practice phase were excluded, as it was assumed that they had not understood the instructions.

#### Concentration

At each measurement point, concentration performance was measured using the Flower Test (Koch et al., 2021). In this test, flowers in different color combinations appear in a row on the tablet screen. For a period of 3 min, children were asked to move the flowers of a certain color combination upwards on the screen with their fingers. The remaining flowers that did not match this description were to be pushed to the bottom. Whenever a row of flowers was completed, a new row appeared. Children were instructed to sort the flowers as quickly and as accurately as possible. The faster the children worked, the more flowers they were given within the 3 min. The concentration indicator, calculated as the difference between the number of correct and incorrect answers, was used for analyses. The higher the concentration indicator, the greater the ability to concentrate. Only cases with a positive concentration indicator were included in the analyses, as a negative concentration indicator suggested that the test instructions were not understood.

### Statistical analyses

All statistical analyses were conducted with the software R (R Core Team, 2023). Before the analyses, data were screened for outliers and multicollinearity. The data were analyzed using linear mixed-effects models with repeated observations nested within children. A random intercept for children was included to account for within-child dependency across measurement occasions. Although children were nested within classrooms, the number of classrooms was small (*N* = 6), and the estimated between-class variance was negligible (≈0). Therefore, classroom-level random effects were not included in the final model. A more complex random-slope specification for time was tested but did not improve model fit and resulted in a singular solution, with the estimated variance of the random slope being zero. Consequently, the final model retained random intercepts for children only.

## Results

### Data preparation and descriptive statistics

At T2 and T3 (but not at T1), the Rosner test (Rosner, 1983) identified values of 3, 4, 5, and 8 points in the ATEM 3–9 as statistical outliers. Inspection of the longitudinal data showed that these very low scores were inconsistent with the respective children’s performance at the previous or following measurement points, at which all four children showed scores within the average range. Because these isolated values were considered unlikely to reflect stable individual differences in emotion understanding, they were excluded from the analyses. For trait mindfulness one outlier was detected at T1 which was also excluded from further analyses due to the same reasons. Descriptive statistics of children’s emotion understanding, breath counting scores and trait mindfulness scores after the exclusion of outliers are displayed in Table 1. Children’s average performance in the emotion understanding test, in the breath counting task and their scores in the trait mindfulness scale increased slightly over time. Data for the breath counting task could only be analyzed for 50 to 53 children at each measurement point because of the restriction of plausible circle durations.

**Table 1:** Descriptive statistics of main variables.

	T1	T2	T3
Emotion understanding			
*N*	66	64	59
Mean (*SD*)	20.24 (5.31)	22.83 (3.35)	23.90 (3.17)
Skew	−1.13	−0.74	−0.20
Kurtosis	0.91	0.20	−0.57
Range	5–28	13–28	16–31
Breath-based state mindfulness			
*N*	53	50	51
Mean (*SD*)	0.57 (0.25)	0.62 (0.24)	0.68 (0.23)
Skew	−1.15	−0.49	−0.83
Kurtosis	−0.61	−0.63	0.09
Range	0.03–1.00	0.03–1.00	0.06–1.00
Trait mindfulness			
*N*	63	66	62
Mean (*SD*)	51.38 (10.22)	54.95 (12.49)	55.29 (12.59)
Skew	−0.40	−0.84	−1.02
Kurtosis	−0.84	0.36	0.56
Range	25–70	17–75	19–74

Table describes the main variables emotion understanding, breath-based state mindfulness, and trait mindfulness in terms of their sample size, mean, standard deviation (SD), skew, kurtosis and range for each measurement point after the exclusion of outliers.

Correlations between all study variables are presented in Table 2. According to Cohens conventions (Cohen, 1988) correlations between emotion understanding and breath-based state mindfulness at the corresponding time points were small to medium sized but did not reach statistical significance. The correlations between emotion understanding and trait mindfulness were near zero at T1 and T3 and of medium size at T2, but none was statistically significant. Associations of each working memory updating and concentration with emotion understanding were small. At T1, girls (*M* = 22.03) performed better in the emotion understanding test than boys [*M* = 18.76, *t*(62) = −2.67, *p* = 0.012]. However, the difference decreased at T2 [*M*_girls_ = 23.43, *M*_boys_ = 22.36, *t*(56) = −1.24, *p* = 0.111] and increased again at T3 [*M*_girls_ = 24.93, *M*_boys_ = 23.13, *t*(51) = −2.10, *p* = 0.041]. Children’s emotion understanding at T1 did not differ significantly based on their DLL status [*t*(64) = 1.62, *p* = 0.111], or their parents’ vocational qualifications [*t*(60) = 1.26, *p* = 0.213]. In addition, neither children’s emotion understanding [T1: *t*(64) = −0.11, *p* = 0.917; T2: *t*(58) = −0.48, *p* = 0.635; T3: *t*(53) = 0.68, *p* = 0.500], nor their breath-based state mindfulness [T1: *t*(53) = 1.30, *p* = 0.200; T2: *t*(51) = −0.42, *p* = 0.680; T3: *t*(55) = 0.70, *p* = 0.486], nor their self-reported trait mindfulness [T1: *t*(61) = 0.60, *p* = 0.554; T2: *t*(64) = −0.21, *p* = 0.834; T3: *t*(60) = 1.10, *p* = 0.274] was associated with their assignment to the intervention versus control group at any measurement point. Accordingly, working memory updating, concentration, and gender were included as control variables in the main analysis, whereas DLL status, parents’ vocational qualifications, and assignment to the intervention versus control group were not controlled for.

**Table 2:** Holm-adjusted Pearson correlations between study variables.

		1	2	3	4	5	6	7	8	9	10	11	12	13	14	15
1	T1 Age															
2	T1 Emotion Understanding	−0.32														
3	T2 Emotion Understanding	−0.21	0.59^***^													
4	T3 Emotion Understanding	−0.07	0.47^*^	0.57^***^												
5	T1 Breath Counting	−0.11	0.30	0.05	−0.01											
6	T2 Breath Counting	−0.08	0.37	0.41	0.30	0.28										
7	T3 Breath Counting	−0.20	0.25	0.25	0.25	0.17	0.39									
8	T1 Trait Mindfulness	0.19	−0.06	0.27	0.25	−0.06	0.32	0.22								
9	T2 Trait Mindfulness	−0.06	−0.06	0.37	−0.02	−0.06	0.04	0.21	0.47^**^							
10	T3 Trait Mindfulness	0.00	−0.06	0.28	0.02	0.03	−0.04	0.08	0.50^**^	0.60^***^						
11	T1 Working Memory Updating	0.02	0.25	0.15	0.03	0.12	0.00	0.01	0.15	0.22	0.21					
12	T2 Working Memory Updating	−0.11	0.23	0.36	0.20	−0.17	0.15	0.14	0.25	0.25	0.16	0.14				
13	T3 Working Memory Updating	0.03	0.13	0.23	0.15	0.10	0.11	0.10	0.20	0.30	0.25	0.29	0.23			
14	T1 Concentration	−0.28	0.16	0.18	0.11	0.15	−0.08	0.06	0.23	0.34	0.30	0.10	0.13	0.07		
15	T2 Concentration	−0.36	0.29	0.26	0.07	0.42	0.17	0.19	0.19	0.31	0.20	0.38	0.07	0.18	0.51^*^	
16	T3 Concentration	−0.34	0.42	0.34	0.12	0.43	0.16	0.20	0.04	0.32	0.03	0.27	0.11	0.23	0.27	0.75^***^

*N* = 39–66; ^*^p < 0.05, ^**^p < 0.01, ^***^p < 0.001.

Table shows the bivariate Holm-adjusted Pearson correlations between the main variables emotion understanding, breath-based state mindfulness, and trait mindfulness and the control variables working memory updating, concentration for all three measurement points and age at T1. The sample sizes for the correlations ranged from 39 to 66, with only the strong correlations within variables and between measurement points reaching statistical significance.

The mean differences in breath counting scores in the intervention group were 0.10 (*SD* = 0.31) between T1 and T2 and 0.01 (*SD* = 0.28) between T2 and T3 compared to −0.05 (*SD* = 0.28) between T1 and T2 and 0.11 (*SD* = 0.26) between T2 and T3 in the control group. For both periods, differences between groups were not significant [T1/T2: *t*(41) = −1.55, *p* = 0.130, T2/T3: *t*(44) = 1.16, *p* = 0.253]. The mean differences in trait mindfulness scores in the intervention group were 5.69 (*SD* = 11.69) between T1 and T2 and −1.20 (*SD* = 12.79) between T2 and T3 compared to 1.67 (*SD* = 10.67) and 2.85 (*SD* = 8.79) in the control group. Again, the groups did not differ significantly [T1/T2: *t*(60) = −1.39, *p* = 0.169, T2/T3: *t*(60) = 1.41, *p* = 0.164].

### Main analysis

A total of 58 children from 6 classrooms could be included in the main analysis. Data from the three measurement points led to 121 observations. Using a multilevel mixed-effects linear model, the effects of children’s breath-based state mindfulness and trait mindfulness on emotion understanding across all measurement points were analyzed, controlling for working memory updating, concentration, age, and gender (see Table 3). Emotion understanding increased significantly over time. Breath-based state mindfulness was positively associated with emotion understanding, whereas trait mindfulness was not significantly related to this outcome. Among the control variables, working memory updating showed a small positive association with emotion understanding. No significant effects were found for age, gender, or concentration. The model explained 24% of the variance in emotion understanding based on fixed effects (marginal *R*^2^ = 0.24) and 67% when including random effects (conditional *R*^2^ = 0.67) (Nakagawa et al., 2017).

**Table 3:** Multilevel mixed-effects linear model on emotion understanding.

	B	SE	DF	*β*	*t*
Intercept	22.293	6.047	68.435		3.687***
Time	1.205	0.526	120.999	0.115	2.292*
Age	−0.072	0.057	65.770	−0.352	−1.275
Gender	1.380	0.826	58.237	0.061	1.670
Working memory updating	0.346	0.174	93.161	0.063	1.994*
Concentration	0.007	0.022	119.053	0.011	0.302
Breath-based state mindfulness	3.131	1.134	91.243	0.093	2.761**
Trait mindfulness	0.033	0.025	115.998	0.080	1.296

B = unstandardized regression coefficient; SE = standard error; DF = degrees of freedom; *β* = standardized regression coefficient. ^*^p < 0.05. ^**^p < 0.01. ^***^p < 0.001.

A simulation-based power analysis was conducted based on the final mixed-effects model. For breath-based state mindfulness, the estimated power was 80.2% (95% CI [77.6, 82.6%]), indicating that the study was adequately powered to detect effects of the observed magnitude. In contrast, the power to detect the observed effect of trait mindfulness was substantially lower (approximately 28%), suggesting that the study was underpowered to detect effects of this magnitude. Although the standardized coefficients were similar in magnitude, the estimates differed in precision, as reflected in the larger standard error for trait mindfulness. Accordingly, statistical power differed substantially between these two predictors.

## Discussion

In the present study, the effects of breath counting skills as an objective behavioral measure of state mindfulness, as well as self-reported trait mindfulness, on emotion understanding were investigated in a sample of third-grade German primary school children across an eight-months period. In the multilevel mixed-effects linear model, breath-based state mindfulness significantly predicted children’s emotion understanding beyond the small but significant effects of working memory updating and time. Although the effect of trait mindfulness was similar in magnitude to that of breath-based state mindfulness, it was not statistically significant. Children’s age, gender, and concentration skills also showed no significant associations with emotion understanding. To our knowledge, the present study is the first examining the unique contributions of breath-based state mindfulness and trait mindfulness to emotion understanding in primary school children. Research on the association between mindfulness and social-emotional competence and relationship skills in children generally is very sparse. In fact, we could not find any previous study that examined the relationship between state mindfulness and emotion understanding or similar constructs in children.

### State mindfulness and emotion understanding in children

This study also addresses the potential bias associated with self-reports of state mindfulness by employing objective measures to assess both state mindfulness and emotion understanding. Therefore, our results on the association between state mindfulness and emotion understanding cannot be directly compared with previous findings. However, our findings are consistent with Feuerborn and Gueldner’s (2019) notion that the concepts of mindfulness-based practices and SEL are to some extent aligned. The results showed that children who were better able to maintain focus on their breathing for a longer period, without being distracted by their thoughts or surroundings, demonstrated greater ability to recognize and understand other’s emotions. The fact that breath-based state mindfulness showed effects beyond the significant effect of working memory updating and the non-significant effect of concentration suggests that the ability to maintain focus on one’s breath is distinct from the ability to temporarily store and update information or to concentrate on a specific task. Moreover, it is in line with the results from Levinson et al. (2014) whose data suggest that breath-based mindfulness is not limited to working memory or sustained attention.

### Trait mindfulness and emotion understanding

The results of the present study are in line with previous studies in which no association between self-reported trait mindfulness and aspects of self-reported emotion understanding (i.e., “interpersonal ability,” “perceiving and understanding emotions,” and “understanding emotions” factors of emotional intelligence) were found in children or adolescent samples (Foster et al., 2018; Mestre et al., 2019; Rodríguez-Ledo et al., 2018). In this study, we extend these findings to a slightly younger sample and to an objective measure of emotion understanding. One possible explanation for the non-significant association between trait mindfulness and emotion understanding is that trait mindfulness may not yet be fully established as a stable trait-like self-concept in primary school children. Although children may be able to engage in mindful attentional states during concrete tasks, reliably reflecting on one’s general level of mindfulness may require more advanced metacognitive and introspective abilities. This interpretation is consistent with concerns regarding the validity of self-report mindfulness measures in younger populations (Brown and Ryan, 2003; de Bruin et al., 2013). Moreover, consistent with Zhuang et al. (2025), state and trait mindfulness were unrelated in the present study, in contrast to the weak but significant correlations reported in adults (Levinson et al., 2014). This may indicate ongoing developmental changes in trait mindfulness and its association with state mindfulness during childhood. Furthermore, emotion understanding may depend more strongly on momentary attentional engagement and present-moment awareness than on generalized self-perceptions of mindfulness. Breath-based mindfulness tasks may therefore capture attentional and meta-awareness processes that are more directly relevant for processing emotional information in social situations. At the same time, the lower statistical power for trait mindfulness indicates that smaller effects may have remained undetected and should therefore not be ruled out completely. Future studies should incorporate both self-report and objective measures of mindfulness and emotion understanding in larger, more diverse samples to examine whether there are systematic differences in the association between mindfulness and social-emotional and relationship competences based on age and method of assessment.

### Intervention effects on mindfulness

Although it was not the focus of the present study, we acknowledge that the Breathing Breaks intervention (von Salisch and Voltmer, 2023; Voltmer et al., 2023) did not lead to a significantly greater increase in breath-based state or trait mindfulness in the intervention group. Mean score differences between T1 and T2 for breath counting and trait mindfulness were higher in the intervention group, but these differences did not reach statistical significance and were also reversed in the period between T2 and T3. The short duration of the intervention phase and irregular or absent practice during the lockdowns caused by the pandemic, especially between T2 and T3, may have contributed to these inconsistent results. However, a study with adult participants demonstrated that performance in breath-based mindfulness can improve with practice (Isbel et al., 2020), and Zhuang et al. (2025) further showed that breath counting performance of children was responsive to breath-focused mindfulness practice. Future longitudinal studies should examine whether an increase in breath-based mindfulness performance is causally linked to improvements in emotion understanding in children.

### Limitations

Despite the strength of using a breath counting task as an objective measure of state mindfulness in children, it is important to acknowledge that evidence supporting the validity of this task in children is currently limited. In addition, despite the known limitations of using a self-report questionnaire to assess children’s trait mindfulness, we were unable to include more reliable measures such as repeated assessments over a longer time frame, for example through ecological momentary assessment. Future studies on mindfulness should follow a multi-method approach for both state and trait mindfulness to increase quality of the data. Also, emotion understanding scores for the children in the present study (*M* = 20.24) were rather low compared to the age groups’ scores in the norming sample (*M* = 23.88 for 8-year-olds, Voltmer and von Salisch, 2021). Emotion understanding was assessed at the end of a test battery that took several hours to complete (with breaks in between), which may have fatigued the children and negatively affected their performance. It therefore remains possible that children’s emotion understanding was underestimated in this study. However, this would apply to all children in the sample and should not influence the association between mindfulness and emotion understanding. Nevertheless, future studies examining the relationship between mindfulness and social–emotional abilities should ensure that the data collection process with children is neither excessively time-consuming nor stressful. Furthermore, social and environmental variables, especially during the pandemic, may have affected children’s development of emotional understanding. Although we found no association with emotion understanding for, e.g., child-reported classroom climate (*r* = 0.01, *p* = 0.878), we cannot rule out the influence of potential confounding variables. Finally, the sample size in the present analyses was relatively small, and sample sizes varied across analyses due to missing data, which may have reduced statistical power. This, together with the narrow age range, limits the generalizability of the findings. Replicating our findings in larger samples with a broader age range would increase confidence in the association between mindfulness and emotion understanding.

### Practical implications

Although caution is needed when interpreting the results due to the small effect sizes, the findings of the present study indicate that practicing mindfulness not only promotes intrapersonal cognitive or affective self-regulation skills in children, as has been shown in earlier studies (Phan et al., 2022), but also interpersonal social-emotional skills. Until now, SEL- lessons on social-emotional learning have often been explicitly included in extensive extracurricular school-based MBIs (Schonert-Reichl et al., 2015; Thierry et al., 2022). However, this study suggests that even basic and very time-effective mindfulness practices like maintaining focus on one’s breath, in which social-emotional content is not explicitly included, may promote interpersonal social-emotional skills. This is particularly relevant for school settings, where limited instructional time and practical constraints often hinder the implementation of more extensive intervention programs. Short and easily implementable mindfulness exercises could therefore represent a feasible low-threshold approach to support children’s social-emotional development within everyday classroom routines.

### Future directions

This study gives first hints that there exists an association between mindfulness and social-emotional skills. By exploring the associations between specific facets of state and trait mindfulness (e.g., observing, acting with awareness and accepting without judgment) and distinct components of emotion understanding (e.g., recognition, Theory of Mind related, knowledge of emotion regulation strategies), future studies could provide a more detailed picture. This could help identify whether the strength of the association with mindfulness varies between basic emotion recognition skills and more advanced emotion understanding. Possible mediation effects of third variables should also be investigated to gain more information about the way MBIs influence social-emotional skills.

## Conclusion

This study addresses the concern that MBIs in schools mainly practice intrapersonal skills, neglecting interpersonal skills that are very important for everyday school life. While MBIs that explicitly incorporate aspects of SEL have already been shown to promote social awareness and relationship skills, the present study showed that higher levels of breath-based state mindfulness in children, as demonstrated by a higher score on the breath counting task, were associated with higher levels of emotion understanding. This indicates that mindfulness practice could positively influence not only intrapersonal but also interpersonal skills, even without incorporating aspects of SEL interventions. Future studies should investigate this relation more thoroughly and consider potential mediating factors.

## Data Availability

The raw data supporting the conclusions of this article will be made available by the authors, without undue reservation.
